# Compendium of Plant-Specific CRISPR Vectors and Their Technical Advantages

**DOI:** 10.3390/life11101021

**Published:** 2021-09-28

**Authors:** Anshu Alok, Hanny Chauhan, Santosh Kumar Upadhyay, Ashutosh Pandey, Jitendra Kumar, Kashmir Singh

**Affiliations:** 1 Department of Biotechnology, Panjab University, Chandigarh 160014, India; anshualok2@gmail.com (A.A.); hannysingh31@gmail.com (H.C.); 2 Department of Plant Pathology, University of Minnesota, Saint Paul, MN 55108, USA; 3 Department of Botany, Panjab University, Chandigarh 160014, India; skupadhyay@pu.ac.in; 4 National Institute of Plant Genome Research, New Delhi 110067, India; ashutosh@nipgr.ac.in; 5 Department of Agronomy and Plant Genetics, University of Minnesota, Saint Paul, MN 55108, USA

**Keywords:** CRISPR/Cas9, Csy4-gRNA, prime editing, multiplexing, base editing

## Abstract

CRISPR/Cas mediated genome editing is a revolutionary approach for manipulating the plant genome. However, the success of this technology is highly dependent on selection of a specific vector and the other components. A plant-specific CRISPR/Cas vector usually consists of a *Cas* gene, target-specific gRNA, leader sequence, selectable marker gene, precise promoters, and other accessories. It has always been challenging to select the specific vector for each study due to a lack of comprehensive information on CRISPR vectors in one place. Herein, we have discussed every technical aspect of various important elements that will be highly useful in vector selection and efficient editing of the desired plant genome. Various factors such as the promoter regulating the expression of Cas and gRNA, gRNA size, Cas variants, multicistronic gRNA, and vector backbone, etc. influence transformation and editing frequency. For example, the use of polycistronic tRNA-gRNA, and Csy4-gRNA has been documented to enhance the editing efficiency. Similarly, the selection of an efficient selectable marker is also a very important factor. Information on the availability of numerous variants of Cas endonucleases, such as Cas9, Cas12a, Cas12b, Casɸ, and CasMINI, etc., with diverse recognition specificities further broadens the scope of editing. The development of chimeric proteins such as Cas fused to cytosine or adenosine deaminase domain and modified reverse transcriptase using protein engineering enabled base and prime editing, respectively. In addition, the newly discovered Casɸ and CasMINI would increase the scope of genetic engineering in plants by being smaller Cas variants. All advancements would contribute to the development of various tools required for gene editing, targeted gene insertion, transcriptional activation/suppression, multiplexing, prime editing, base editing, and gene tagging. This review will serve as an encyclopedia for plant-specific CRISPR vectors and will be useful for researchers.

## 1. Introduction

Clustered regularly interspaced short palindromic repeats (CRISPR) and its associated Cas protein (CRISPR/Cas) has revolutionized the area of plant genome engineering. It is comparatively precise, easy to design, low-cost as compared to other approaches, such as Zinc-finger nucleases (ZFNs) and transcription activator-like effector nucleases (TALENs) [1,2]. Identification of diverse Cas proteins such as Cas9, Cas12a (Cpf1), Cas12b, Cas12e (CasX), and Cas12j (Casɸ) with different PAM specificity has led to the development of numerous editing vectors specific for plant genome modifications [3,4]. These tools have been frequently applied to knockout the desired gene, gene correction, site-specific transgene incorporation, etc., in a flexible manner in various plant species [5,6,7].

CRISPR-Cas mediated genome engineering requires an endonuclease (Cas9, Cas12a, or Cas12b, Cas12j) and a guide RNA (gRNA) as the essential components. The gRNA first recognizes the target site, which is followed by the cleavage of double-stranded DNA by the endonuclease. The double-strand break (DSB) is subsequently repaired by nonhomologous end-joining (NHEJ) or by homology directed repair (HDR) [8,9]. HDR uses a DNA template and repairs DSB without any error. Target specificity depends upon the first 20 nucleotides (nt) of gRNA, also known as a spacer, which recognizes the complementary target sequence (protospacer) within the genome. The selection of 20 base pairs (bp) specific and unique target sequences is essential for efficient and precise editing. Furthermore, the presence of a protospacer-adjacent motif (PAM) is essential during target selection [10,11]. PAM sequences for Cas9, Cas12a, and Cas12b are 5’NGG3’, TTTV, and ATTN, respectively (N could be any nucleotide and V is A, C, or G). Cas9 is an RNA-guided monomeric DNA endonuclease that is an essential component of the type-II CRISPR system found in some bacterial genomes. It creates DSB at the target sequence of the genome, which may lead to mutations. The DSB is usually repaired by an inherent plant cell repair mechanism, which sometimes inserts frameshift mutations by deletion or addition of nucleotides [12]. The Cas9 protein has a globular recognition (REC) and a small nuclear (NUC) lobe. The REC lobe consists of a bridge helix, REC1, and REC2 domains, whereas the NUC lobe comprises the RuvC, HNH, and PAM-interacting domains [13,14]. RuvC nuclease domain cleaves the target strand while the complementary strand is cleaved by HNH nuclease, which results in a DSB [8,14]. The size of the *Cas9* gene isolated from *Streptococcus pyogenes*, *Neisseria meningitidis*, *Streptococcus thermophilus*, and *Treponema denticola*, range from 3.25 to 4.6 kilobase pairs (kb) and recognize diverse PAM sequences [15]. The length of the *Cas9* gene in CRISPR vectors engineered with *S. pyogenes* and *Staphylococcus aureus* are 4.1 and 3.1 kbp, respectively [14,16]. Many orthologs of *Cas9*, *Cas12a*, *Cas12b*, and *Cas12j* genes have been identified, which vary in size and recognition sites (Table 1). Alteration in an amino acid of RuvC (D10A) or HNH (H840A) nuclease domains of Cas9 leads to partial function loss, which causes the nicking of one strand of DNA in place of DSB. This modified Cas9 is known as “nCas9” [8]. However, such mutations in both RuvC and HNH nuclease domains resulted in dead Cas9 (dCas9) that could only bind to the target site [10]. A variant *Streptococcus pyogenes* (SpCas9) had the lowest tolerance for mismatched target sequences, whereas SpCas9-NG has the broadest PAM compatibility [17]. Two engineered Cas9 variants, namely SpG and SpRY, were found to recognize a broader range of PAM sequences in rice [18].

**Figure 1:**
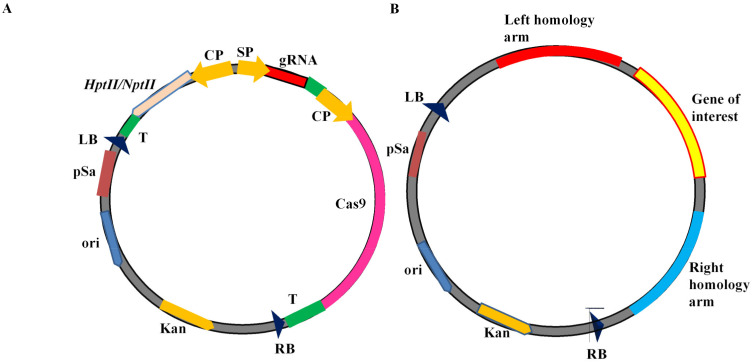
Schematic diagram of CRISPR vector. (**A**) A typical CRISPR/Cas9 plant transformation vector for gene editing; (**B**) typical donor vector for targeted gene insertion; (CP) constitutive promoter such as Ubi, Act, and CaMV35S; (SP) small RNA promoter such as U6 and U3 promoters, (T) terminator such as NosT, (LB) left border of T-DNA, (RB) right border of T-DNA, and (HptII/NptII) selection genes.

Various components of the CRISPR-Cas editing tool have been assembled into desired cloning and binary/plant transformation vectors that are usually known as CRISPR vectors [5,26,27]. A typical CRISPR/Cas9 plant transformation vector consists of a *Cas9* gene and a gRNA regulated by appropriate promoters (Figure 1). Cloning vectors carrying Cas9 and gRNA have been used for genome editing in protoplast, and successive regeneration into transgene-free genome-edited plants. Purified recombinant Cas9 protein and in vitro transcribed gRNA have also been successfully applied for genome editing in numerous plants such as potato, wheat, maize, etc. [28,29,30,31]. This method is known as a vector-less or DNA-free editing tool and is directly delivered within the plant cells using gold particle or PEG-mediated protoplast transformation. Several types of vectors have been designed for specific objectives using the new Cas proteins and knowledge about Cas protein engineering. These include editing, interference, activation, knock-in, promoter bashing, methylome-editing, base editors, transposases/recombinases, multiplex genome editing, and prime editors, etc. [7,32,33,34].

In the current review, we have discussed all the technical aspects of each component and their potential contribution to improved editing frequency in plants. Furthermore, the categories of CRISPR vectors, their role in plant genetic engineering, and technical details have been comparatively summarized, which will be highly useful to researchers working in the area of plant genome engineering

## 2. Size of gRNA Is Important for Efficient Cas9 Endonuclease Activity

The gRNA used in CRISPR vectors is usually a fused truncated sequence of crRNA and tracrRNA [35]. The synthetic gRNA mimics the natural crRNA-tracrRNA hybrid. The size of the most frequently used gRNA in CRISPR vectors is 76 bp [6,32,36,37]. Initially, various sizes of tracrRNAs were fused to form variants of gRNA, i.e., gRNA (+48), gRNA (+67), and gRNA (+85). An in vitro cleavage assay demonstrated that the gRNA (+48) is the minimal region for DNA cleavage by the Cas9 [8]. However, the increased sizes of gRNA, i.e., gRNA(+67) and gRNA (+85), significantly improved the Cas9 cleavage activity in vivo [35]. A typical CRISPR vector consisted of a 400–500 bp long gRNA cassette, which consists of RNA polymerase III promoters, gRNA, and Pol III terminator [32]. Cas12j or Casɸ require 41–49 bp gRNA, which consists of 16–24 bp target spacer sequence fused with 25 bp crRNA [4].

## 3. Polycistronic-tRNA/Csy4-gRNA Is a Need for Editing of Multiple Targets with a Single Construct 

Initially, different promoters and terminators were used to regulate the multiple gRNAs within the same vector during multiplex genome editing, which led to a significant increase in the size of the vector [37]. Assembling several gRNA cassettes within a single vector was a challenge due to the constraint of the plasmid vector capacity and delivery method into plant cells [32]. This was resolved using linker sequences during the assembly of multiple gRNAs. These linker sequences are recognized and chopped by either **C**RISPR/Cas **S**ubtype **Y**pest (Csy)-type endoribonuclease 4 (Csy4) or transfer RNA (tRNA) processing enzymes within cells [38]. Furthermore, these multiple gRNAs flanked by Csy4 recognition sites are regulated by a single promoter, which reduces the size of the vector. Csy4 binds and cleaves the repetitive linker/spacer sequences [39]. The size of the *Csy4* gene is 585 bp, which encodes a 21.4 kDa protein with histidine in its active site. A separate promoter can regulate the *Csy4* gene in a vector, or it can be fused along with *Cas9* under the same promoter. *P. atrosepticum* is another source for Csy4 nuclease and its associated 28 nt recognition RNA sequence [40]. Single transcripts containing repeated *Csy4* spacer-*gRNAs* are cleaved into multiple individual gRNA within the cell [39]. The *Csy4*-*gRNAs* synthetic gene is regulated by pol II promoter and successfully transcribed within the cells [38,41].

Glycine tRNA has also been used to construct synthetic polycistronic–*tRNA*–*gRNA* genes. Endogenous tRNA-processing enzymes, RNaseP and RNaseZ, cleave the transcripts having tRNA-gRNAs in the plant cells. These tRNA-processing enzymes are universal and present in all the plants; therefore, there is no need to introduce an extra *RNase* gene with a CRISPR vector [36,42]. Furthermore, the tRNA acts as an enhancer and boosts the Pol III transcription efficiency, which is an additional advantage with this system [43]. The 77 bp-long tRNA spacer sequence has been used to deliver multiple gRNA in *Arabidopsis* and rice [42].

## 4. Importance of Promoter Regulating *Cas9* and gRNA towards Editing Efficiency

The promoter regulating the expression of the *Cas9* gene is crucial for efficient genome editing. Mostly ubiquitin and CaMV35S promoters have been used to regulate the *Cas9* gene in the CRISPR/Cas9 toolkit. The strength of a promoter can be correlated with the mutation frequency at the target site. In *Zea mays*, the ubiquitin promoter showed 20% more mutagenesis frequency than the CaMV35S [44]. In *Arabidopsis*, the plant codon-optimized *Cas9* was regulated under the UBQ10 promoter, which showed higher expression in early embryos and later stages in developed plants [45]. The vectors pRGEB31 and pRGEB32 contain CaMV35S and rice ubiquitin promoters, respectively, for regulating *Cas9* gene expression and rice snoRNA pol III (U3) promoter to regulate gRNA [32,36]. The vector HBT-pcoCas9 consists of the plant codon-optimized *Streptococcus pyogenes Cas9* gene under the regulation of a strong constitutive hybrid 35SPPDK promoter. This promoter has potato IV2 intron, which overcomes the alleviated problems associated with the cloning of the *Cas9* gene in *E. coli* [46].

Small nuclear RNA promoters such as U3 and U6 have been frequently used to regulate gRNA expression in CRISPR vectors. The U6 RNA polymerase III promoter expresses constitutively and permits the mRNA to bypass the post-transcriptional modifications. Therefore, the gRNA transcript is retained inside the nucleus [47,48]. Moreover, the U6 promoter requires a guanosine nucleotide to start transcription and works at target sites with GN19NGG and GH20NGG sequences (N: any nucleotide; H: A/T/C) [10,49]. The T7, T3, and SP6 promoters also require initiating guanosine nucleotide during in vitro transcription. Therefore, the RNA pol II promoter has been frequently used in the majority of CRISPR vectors. Further, the H1 pol III promoter can also initiate transcription with any nucleotide at the target site [49]. The AtU3 promoter is more efficient for gRNA expression in dicot plants, whereas the OsU3 promoter is more efficient for gRNA expression in cereals and monocot plants.

## 5. CRISPR On/Off Strategy Uses Inducible Promoter

CRISPR/Cas is a dominant genetic engineering tool, however uncontrolled expression of Cas endonuclease causes various off-target editing within a genome. The use of inducible promoters to regulate *Cas9* and *gRNA* showed efficient editing and reduces off-target effects within the genome [50]. In rice, heat shock inducible promoter was used for CRISPR/Cas9 mediated controlled gene editing. A soybean heat shock protein inducible promoter regulating Cas9 showed a 50–63% mutagenesis rate in transgenic line under heat stress, while a low mutagenesis rate was noticed without heat stress. The transgenic rice lines edited with inducible promoter resulted in undetectable or lower off-target effects as compared to lines edited with a constitutive promoter [51]. A chemical-based inducible promoter system, XVE inducible, was developed by fusing the DNA-binding repressor domain (LexA), the acidic transactivating domain (VP16), and a regulatory region of the human estrogen receptor and successfully applied in plants. The XVE promoter is strictly induced by estradiol in plants [52].

## 6. Plant Selectable Markers Are Needed

Plant selectable marker genes are required during the selection and regeneration of transformed explants. Although one can knock-in/out target genes without the use of a selectable marker, it requires unnecessary large-scale screening. Vectors primarily contain a bacterial selectable marker for screening during the cloning process in *E. coli.* Additionally, a plant selectable marker is necessary for the screening of transformed plant cells. Most CRISPR/Cas9 plant transformation vectors contain *NptII*, *HptII*, and *Bar* genes, which confer resistance against kanamycin, hygromycin, and basta, respectively. For example, pRGEB31, pRGEB32, and pHSN401 provide hygromycin selection, whereas pBUN411 and pBUE411 contain basta selection [32,36,37]. The plant selectable markers are absent in CRISPR vectors used for transient transformation in protoplasts such as pRGE31. However, the regeneration and screening of genome-edited plants grown without selective media from protoplasts are not feasible and tedious in many plant species. Choosing the right selectable marker along with CRISPR/Cas9 components is an essential factor for the efficient regeneration of transgenic plants.

## 7. Selectable Marker and Plasmid Backbone Affecting Transformation Efficiency

The plant selectable markers present in the CRISPR vector and plasmid backbone are very important for the selection of specific plant species. A highly efficient selection system will allow researchers to generate more transgenic lines with increased mutation frequency. The promoter regulating the selectable marker gene is also an important parameter for efficient transgenic plant selection. Predominantly, the selectable marker genes such as *NptII* or *HptII* have been regulated under CaMV35S or NOS promoters. The *NptII* gene regulated under the NOS promoter was reported to be highly efficient for tomato [53,54]. A double enhancer CaMV35S promoter has been used in pPZP, pCAMBIA, pCGN, pGreen and pGPTV vectors. The strength of the CaMV35S promoter is comparatively less in monocot as compared to dicot plants [55]. Moreover, the insertion of CAT-1 intron within the *HPTII* gene increased the transformation efficiency 2.5-fold in rice and barley. For example, the pBRACT vector having the CaMV35S promoter regulate an intron-containing *HPTII* gene was found to be highly efficient in barley [56,57]. However, the highest (18%) transformation efficiency was achieved in wheat with the pGGG vector as compared to pAGM (12%) and pBRACT (5%). The rice actin promoter regulating the *HPTII* gene is present in pGGG and pAGM vectors whereas the CaMV35S regulating the *HPTII* gene is in pBRACT. The vector backbone also showed a significant effect on wheat transformation efficiency, which was 5% in pBRACT, 12% in pAGM, and 18% in pGGG [58]. The CaMV35S or rice actin promoters regulating the *HPTII* selectable marker gene having the CAT1 intron showed a significant difference in wheat transformation efficiency. Although the mechanism is not fully understood, some introns such as CAT1 increase the expression of the gene, which suggests the existence of an intron-mediated enhancement mechanism.

In fact, the citrus genotypes, namely pineapple sweet orange and Carrizo citrange, showed higher transformation efficiency of 6.7% and 7.2%, respectively, using the multi-auto-transformation vector. The transformation efficiency increased further to 13–30% for both genotypes with the *PMI* selectable marker, which suggests a crucial role of selectable marker genes in transformation and genome editing [59].

## 8. Origin of Replication and Its Importance during Molecular Cloning

The presence of an origin of replication (ori) region in CRISPR vectors defines its ability to replicate either in *Agrobacterium* or in *E. coli* or both. The most used ori for *Agrobacterium tumefaciens* are pRK2, pVS1, and pSa1; for *Agrobacterium righogenes* is pRi, and for *E. coli* are pRK2, ColE1, and pUC [60]. ColE1, pBluescript, and pUC maintain about 15–20, 300–500, and 500–700 copies per cell. The CRISPR vectors should be carefully analyzed before preceding the experiment by keeping the *ori* in mind. The CRISPR vectors such as pBUN41, which was developed using the pGreen backbone, cannot replicate in *Agrobacterium*. To replicate the pBUN41 vector in *Agrobacterium*, it needs the pSoup plasmid from the same strain [37]. The pGreen-derived CRISPR vectors do not have RepA and Mob functions, which makes them smaller in size [61]. The pSoup vector delivers replication functions in *Agrobacterium* for pGreen-based CRISPR vectors. The best and most frequently used CRISPR vectors are derived from pCAMBIA, which contain two different ori for *Agrobacterium* and *E. coli.*

## 9. CRISPR/Cas Toolkits for Plants

There are different types of CRISPR/Cas tools available for plant genome engineering. These tools may be broadly categorized into two groups: Cas/sgRNA vectors or Cas/sgRNA ribonucleoprotein complexes.

### 9.1. Cas/sgRNA Ribonucleoprotein Complexes as Editing Tools

Vector-less genome editing in plants using Cas9, Cas12a, Cas12b, and Cas12j can be achieved by two methods. In the first method, the gRNA and Cas endonuclease are transcribed in vitro. Then, they are coated with a carrier such as gold or silver particles and delivered into the plant cells. For in vitro transcription, gRNA and Cas endonuclease are regulated by a T7 promoter and then applied for bombardment into calli [62]. In the second method, gRNA transcripts and purified recombinant Cas protein are delivered into plant cells through PEG or bombardment. Vector-less genome editing has been successfully tested in *Arabidopsis*, tobacco, lettuce, rice, grapevine, and apple [28,30]. In maize, these complexes were bombarded into embryos, and transgene-free mutants were recovered [29].

The main advantage of Cas/sgRNA ribonucleoprotein complexes is that they do not require a vector and, therefore, no insertion of the transgene. These complexes can directly be delivered into plant regenerative tissues utilizing either PEG, nanoparticles, or bombardment methods. The transgene-free edited mutant crops will be more acceptable and probably have less issues with regulatory and ethical barriers. Transgene-free editing of mushrooms using ribonucleoproteins showed less browning as compared to wild type, and it also passed the United States regulatory barrier [63]. The drawback of ribonucleoproteins complexes is that they cannot be delivered by *Agrobacterium*-mediated genetic transformation, which is more efficient and cheaper. Another disadvantage is a large number of screenings for finding mutants, which is laborious and time consuming.

### 9.2. Knock-Out CRISPR Vectors

**Figure 2:**
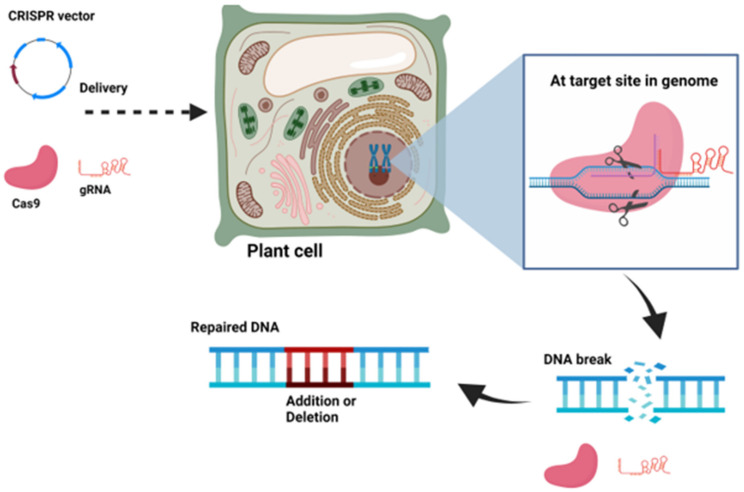
Schematic illustration of CRISPR vector and the mechanism of action in the plant cell and genome. The figure was created with BioRender.com.

These vectors consist of a *Cas* gene, which is regulated by a constitutive or tissue-specific promoter. The gRNA usually has a restriction enzyme site to create an overhang for the cloning of the spacer sequence. For example, pRGEB32, pHSE401, and pBUN411 have a *BsaI* site [36,37]. A few vectors are also available with golden gate assembly for spacer sequence cloning. CRISPR vectors for editing along with their size and selectable marker are listed in Table 2. After successful delivery and integration in the plant genome, co-expressed gRNA identifies the target sequence and Cas9 introduces a DSB at the target site (Figure 2). A few vectors containing nCas9 (mutated Cas9) generate ssDNA breaks (Nick) at the target site in place of DSB produced by the wild-type enzyme [9]. Since nCas9 only nicks the genomic DNA, it requires two different targets within the same gene (one on sense and another on antisense strand) to create DSB. For example, pHSN501, pBUN501, pDGE77, and pYPQ159 vectors contain nCas9. Two adjacent, opposite strand nicks can cause a DSB and trigger error-prone NHEJ repair. These CRISPR vectors reduce off-target binding because they usually require two gRNAs. A few CRISPR vectors consist of catalytically inactive Cas9 (dCas9) fused with FokI nuclease [21]. Two different targets within the same gene, each guided by a unique gRNA, are required to create DSB by dimerization of FokI nuclease fused with dCas9.

The advantage of the CRISPR vector over previous editing tools is that it is easy to design and has better efficiency. The major drawback of CRISPR vectors is the off-target effect within the plant genome. The nonspecific and unintended alteration within the genome is known as off-target genome editing, and this happens due to the close resemblance of gRNA with non-targeted DNA. However, the use of engineered Cas9, such as dimerization of FokI nuclease fused with dCas9, showed less off-target effects. There are a few possible ways to reduce off-target effects. For example, high GC content in gRNA, mismatch length, truncated gRNA, and chemical modification of gRNA help minimize off-target. The GC contents of 40–60% in the gRNA help in reducing off-target activity. Similarly, a mismatch of less than 3 bp in gRNA minimizes the chance of off-target editing.

### 9.3. Knock-In CRISPR Vectors

**Figure 3:**
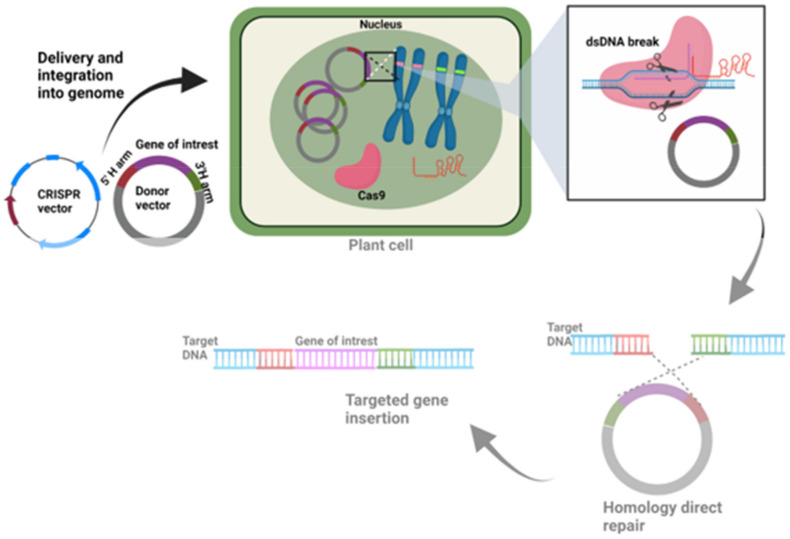
Knock-in CRISPR vector along with a donor vector and its mechanism of action in the plant cell during targeted gene insertion. The figure is created with BioRender.com.

CRISPR-Cas mediated gene correction or knock-in of the desired gene at the target site within the genome significantly increases the application. Cas endonucleases create DNA breaks, which are repaired by either the NHEJ or the HDR repair pathways. NHEJ basically joins the broken DNA ends, which frequently leads to insertions and deletions. However, HDR needs a donor DNA template, a sister chromatid in nature, or an external DNA in the laboratory, which has similarity with the broken DNA region. CRISPR-Cas-mediated gene correction or knock-in requires an exogenous donor template [67]. CRISPR knock-in vectors contain a desired donor template in addition to gRNA and Cas endonuclease. Donor DNA consists of three regions: left and right homology arm and middle sequence (Figure 1B). The donor template is delivered along with the CRISPR-Cas in the same vector or using a different vector. The delivery of gRNA, Cas endonuclease, and donor DNA into the cells initiates the process by cutting the DNA at the target site by Cas endonuclease, which is followed by HDR-mediated DNA repair using donor template (Figure 3). For example, pTC150 and pTC217 are donor template vectors constructed to insert the constitutive promoter CaMV35S before the *ANT1* gene start codon in the tomato genome to regulate the expression of the *ANT1* gene. These vectors consist of sgRNA and Cas9 targeting the *ANT1* locus and donor template. The donor template contains the Pnos:NptII and CaMV35 promoter flanked by 5’ and 3’ homology arms [67].

### 9.4. CRISPRi Vectors

**Figure 4:**
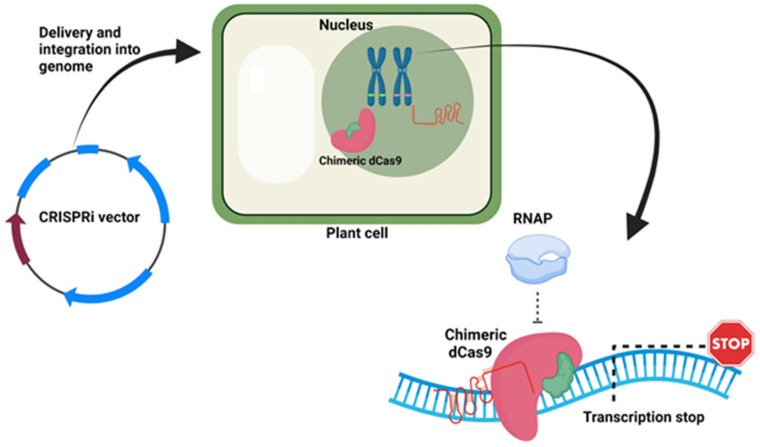
CRISPRi vector and its action in the plant cell. The figure was created with BioRender.com.

CRISPR interference (CRISPRi) vectors are used to interfere with the transcription of the target gene. In CRISPRi, a complex of gRNA and catalytically inactive Cas or dead Cas (dCas) bind the transcriptional start site (TSS) of the target gene and act as an artificial transcriptional suppressor. The first-generation CRISPRi system showed a moderate suppression of the transcription. Whereas, in the second generation, in which dCas9 fused with transcriptional repressor showed better transcriptional suppression [72]. Upon delivery in the plant cells, gRNA directs the binding of the dCas9 repressor to the target site, which ultimately knocks down the gene expression (Figure 4). Examples of CRISPRi vectors are pYPQ153, pHSN6I01, and pdCas9 (GB1079), which can be used in plants [37,71,72]. The potential target for these vectors could be the promoter regions, regulatory regions, and non-coding regions. 

### 9.5. CRISPRa Vectors

**Figure 5:**
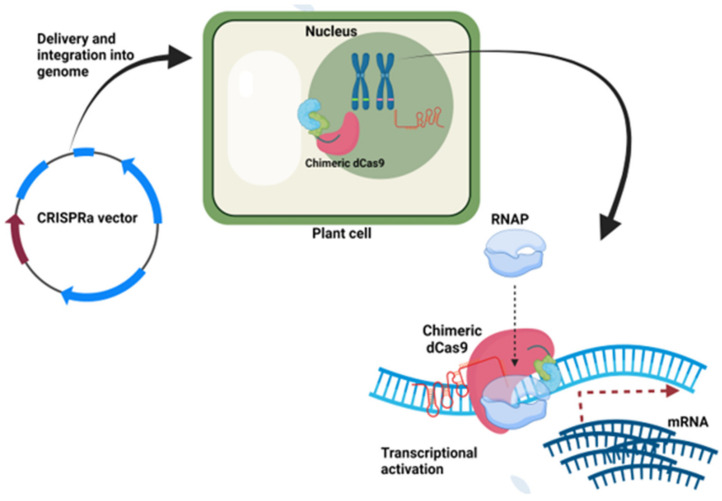
CRISPRa vector and transcriptional activation in the plant cell. The image was created with BioRender.com.

CRISPR-mediated transcriptional activation (CRISPRa) is a mechanism where a complex of the gRNA and effector fused dCas9 binds to the promoter region of the target gene, which results in enhanced transcription. Initially, the most used effector molecules in CRISPRa were transactivators such as VP64, p65, and p300. VP16 is a well-known transcription activator from the herpes simplex virus, whereas VP64 is a tetramer of VP16. p65 is another transactivator, which has an activation domain of the NF-kappa B factor. The second-generation CRISPRa system includes aptamer-specific proteins such as MCP, PCP, and Com that are fused with transactivators. The second-generation CRISPRa system is more powerful and showed higher transcriptional activation as compared to the first generation. The CRISPRa vector contains a dCas9 fused with a transcriptional activator gene, gRNA, and a plant selectable marker gene (Figure 5). For example, pYPQ152, pHSN6A01, pBUN6A11, pdCas9 (GB1079), and pD10AH840AhCas9 (GB1041) vectors are available for plant transformation with different selectable markers [37,71,72]. 

### 9.6. CRISPR Vectors for Visualization

A catalytically inactive Cas9 (dCas9) fused to a fluorescent protein (FP) can be used to visualize specific genomic loci using fluorescent microscopy in living cells [83]. It only requires a gRNA sequence to direct the binding of the dCas9-FP fusion protein to the target location. The potential target locations can be unique or repetitive regions. Furthermore, a single or two different vectors can carry gRNA and dCas9-FP. For example, in tobacco, two separate vectors carrying a dCas9-GFP and a gRNA, separately, targeting the tobacco telomeric region were used to visualize telomere repeats using transient transformation. In *Arabidopsis*, the telomeric region was visualized after stable transformation using these two vectors [74].

### 9.7. Virus-Based CRISPR Vectors

RNA and DNA viruses have also been used for carrying the main two components (gRNA and Cas9) of the CRISPR/Cas system. For example, the RNA2 of the tobacco rattle virus (TRV; an RNA virus with a bipartite genome, RNA1 and RNA2) was used to carry gRNA [84]. However, the carrying capacity of TRV is limited and cannot deliver the whole CRISPR/Cas system. In addition, the RNA virus cannot deliver a DNA repair template [85]. Due to these limitations, DNA viruses such as geminiviruses are frequently used to develop efficient virus-based CRISPR/Cas vectors. For example, pTC217, pTC223 [67], pWDV1-CR [86], pLSLGFP, and pLSLC vectors carrying the green fluorescent protein gene, Cas9, and synthetic gRNA have been developed and used successfully. pLSLC is a geminivirus-based CRISPR vector system, which is derived from the bean yellow dwarf virus. The replication of geminiviruses starts upon binding of the Rep protein to the large intergenic region (LIR) of the viral genome. pLSLC consists of CRISPR/Cas components along with the small intergenic region (SIR) and a gene encoding Rep/RepA, and direct duplications of the LIR flanking sequences [85].

### 9.8. CRISPR Vectors for Transient Expression

These types of vectors are small and are transiently delivered to the protoplast. These vectors have the advantage of not integrating into the genome and thus the genome-edited plants using this technology are considered non-transgenic plants. These vectors are generally delivered to plant cells using either biolistic or protoplast transformation. For example, pRGE31, pRGE32, and p35SPPDK-pcoCas9 are the vectors that are used for transient expression [32,36,46].

### 9.9. CRISPR Cloning/Assembly Vectors

There are several cloning vectors available, which can be used for assembling multiple gRNA followed by transfer to the destination vector. For example, pCBC-DT1T2 is used to assemble two gRNAs within an expression cassette and is regulated by the U6 promoter of *Arabidopsis* (AtU6) [37]. The plasmid pChimera and pEn-C1.1 contain the AtU6 promoter along with sgRNA and are used for classical cloning and gateway cloning into pCAS9-TPC and pDe-CAS9-D10, respectively [87]. In another example, the pYPQ131 to pYPQ138 series vectors have been used for the cloning of eight different gRNA, respectively. Furthermore, the pYPQ142 is used to assemble two guide RNAs, pYPQ14 for three, etc., and pYPQ148 can accommodate eight gRNAs [71].

### 9.10. Base Editing Vectors

The base editing tool empowers precise nucleotide substitutions in the target gene without interruption of the gene in a programmable way [88]. This tool has been proficiently applied in the model plant as well as several crops, including *Arabidopsis*, rapeseed, rice, wheat, and maize [88,89,90,91]. The base editor uses a chimeric protein having catalytically dead Cas9 and a cytosine or adenosine deaminase domain, which converts one base to another. Base editing requires an enzyme able to deaminate adenine or cytosine at the target DNA, in addition to Cas9. Therefore, the base-editing vector consists of a chimeric open reading frame made up of Cas9 and gene encoding cytidine deaminase (C-to-T) or adenine deaminase (A into G, or A–T into G–C). Cytosine base editing (CBE) vector replaces G-C with A-T bases, whereas adenine base editor (ABE) vector converts A-T into G-C [89]. For example, the vectors pH-nCas9-PBE and pnCas9-PBE consist of apolipoprotein B mRNA-editing enzyme, catalytic polypeptide–like (APOBEC) cytidine deaminase [88], whereas the vectors pJY-RpABE and pFH45 are ABE vectors [90].

### 9.11. Prime Editing Vectors

The prime editor is a chimeric protein of nCas9 and modifies reverse transcriptase. It requires multipurpose pegRNA as a gRNA, which contains a template for reverse transcriptase and a primer binding site at the 3’ end [34]. The reverse transcriptase domain uses a nicked strand of DNA as a primer to synthesize a template with an edited DNA flap. Generally, the gRNAs have a spacer sequence that hybridizes to the complementary target DNA. However, the pegRNA contains a few new nucleotides that substitute the nucleotides in target DNA and is used as an extension during the DNA repair [92]. Nicked DNA containing a 3′-hydroxyl group is used to prime the reverse transcription and extension of the pegRNA directly into the target site [34]. This newly emerged CRISPR technology has enabled insertion, deletion, and point mutations and does not require DSB or donor template for repair. Prime editing can be performed using either of the three systems, PE2, PE3, and PE3b. The PE2 comprises an nCas9 (H840A) fused with an engineered Moloney murine leukemia virus reverse transcriptase (M-MLV RT) and a pegRNA. M-MLV RT is an RNA-dependent DNA polymerase, which can synthesize cDNA from long messenger RNAs (>5 kb) [93]. PE3 enhances the nicking of single gRNA to cut the non-edited strand, which enables DNA repair. PE3b, a variant of PE3, recognizes a nicking sgRNA that specifically targets the edited nucleotides which in turn decreases the indel product level by restricting the nicking of the non-edited strand till the complementary strand gets converted into the edited nucleotide [34,92]. In rice, PE2 and PE3 have been efficiently utilized and showed optimum prime editing at the target sites [91].

Prime editing vectors consist of the backbone of a binary vector, pegRNA, and nCas9-RT expression cassettes. For example, pH-nCas9-PPE3 is used for *Agrobacterium*-mediated rice transformation. All the essential components of pH-nCas9-PPE3 were cloned into the backbone of pHUE411 using a ClonExpressII One Step Cloning Kit [92]. The vector PPE3-V01 (Plant Prime Editor 3-Version 1) consists of nCas9-RT regulated by ZmUbi1, pegRNA regulated by OsU3, and a single gRNA regulated the OsU6-2 promoter [91].

## 10. Basis of Choosing the Right Vector

The selection of a CRISPR vector is a very initial and crucial step for plant genome editing. The following parameters are needed to keep in mind during the selection of the CRISPR vector. First, determining the purpose of the editing is important. If there is a requirement to knock out the gene function, then the knockout CRISPR vector must be selected. Moreover, if there is a need to boost the expression of the target gene then the CRISPRa vector must be selected. The second most important criterion is the backbone of the vector and the selectable marker, which will depend upon the plant species. Promoters regulating the expression of the gRNA and Cas endonuclease are the third most important criteria. The following parameters should be considered during the selection of CRISPR vectors.

(i) PAM: which PAM (NGG or NTT or NAG, etc.) is present in the target gene? The type of PAM sequence present in the target sequence will determine the Cas endonuclease and the vector carrying it.
(ii) gRNA: single or multiple?
(iii) Copy number of CRISPR vector: high or low?
(iv) Cloning sites and strategies: restriction enzyme based, golden gate assembly, entry-destination based cloning, and ligation independent cloning, etc.
(v) Antibiotic resistance for bacterial and plant selection.

## 11. Cloning Strategies in Vectors Derived from Different Backbone

Traditional cloning strategies are most frequently used to generate the CRISPR vector backbone. The essential components such as small RNA promoter, synthetic gRNA, constitutive/tissue-specific promoter and Cas endonuclease are cloned together in one plasmid using traditional cloning methods, which uses various restriction enzymes, ligation, bacterial transformation, and confirmation by sequencing. However, due to recent advancements, most of the available CRISPR vectors need only cloning of a protospacer region into the gRNA, such as pRGEB31, pHSE401, and pDIRECT_26H, etc. In recent studies, type IIS restriction enzymes are used most frequently for cloning a gene-specific protospacer adjacent to gRNA in a CRISPR vector. Moreover, the multiple gRNA are cloned into a cloning vector using type IIS restriction enzymes under different small RNA promoters. Then, the full expression cassette is PCR amplified and ligated into the final binary CRISPR vector. Multiple gRNA expression cassettes can be introduced to the CRISPR vector carrying Cas9 with the help of Gibson or Golden Gate Assembly [94].

In another strategy, the entry and destination vectors are used. The entry vectors contain Cas9 or gRNA or both. This type of vector requires LR or BP clonase reaction between the entry and destination vector. A gene-specific protospacer is inserted into an entry clone known as pOs-sgRNA using restriction digestion and ligation. Later, the gRNA cassette is cloned into the destination vector, pH-Ubi-cas9-7 having Ubi:Cas9 using LR clonase. This CRISPR vector is highly efficient in rice [95]. Cloning in the traditional CRISPR vectors requires restriction digestion, ligation, transformation, extensive screening of positive clones which are exhaustive and time consuming. Due to all these constraints, researchers came up with ligation-independent cloning, which has proven to be the most effective. Ligation-independent cloning combines In-Fusion® HD cloning and ccdB-based negative selection. In this system, cloning of multiple gRNA can be achieved directly into the CRISPR vector in a single step [96].

## 12. Conclusions

Recent advancement in the area of plant genetic and metabolic engineering requires user-friendly genome engineering tools. CRISPR/Cas endonuclease-mediated genome engineering has become an ultimate molecular tool in plants. Many of these CRISPR tools are being adopted in various plant species to improve their nutritional and medicinal value [97,98,99,100]. The success of genome editing using CRISPR/Cas9 is highly dependent upon the method of delivery and vector selection. Factors such as selection of promoter to drive the expression of Cas9 and gRNA, the size of gRNA, Cas9 variants, polycistronic *tRNA-gRNA*, polycistronic *Csy4-gRNA*, and vector backbone, etc., are the major components for designing targeted genome editing experiments. Furthermore, the use of polycistronic-tRNA/Csy4-gRNA has enhanced the editing frequencies. Various modifications in Cas endonuclease and the availability of numerous Cas variants have enabled the designing of diverse CRISPR vectors for a broader utility. Transposon-encoded RNA-guided nucleases, known as OMEGA, might be a strong potential candidate for developing new tools in the near future [99]. The newly discovered Cas endonucleases such as Cas ɸ and CasMINI which are smaller in size will be more useful for genetic engineering. The diverse use of CRISPR such as editing, nicking, base editing, targeted gene insertion, transcriptional activation/suppression, multiplexing, prime editing, and gene tagging opens various new avenues for plant scientists, which would ultimately help in developing designer crops to feed the increasing world population.

## Figures and Tables

**Table 1:** List of different Cas endonuclease variants and their recognition sites.

Cas Endonuclease	Size	Bacterial Source	PAM Recognition Site	References
SpCas9	4104 bp	*Streptococcus pyogenes*	3’ NGG	[19]
xCas9 3.7	4140 bp	*Streptococcus pyogenes*	NG, NNG, GAT, and CAA	[17,20]
SpCas9-NG		*Streptococcus pyogenes*	NG	[17]
SpRY		*Streptococcus pyogenes*	PAM-less	[18]
SpG		*Streptococcus pyogenes*	NGD	[18]
StCas9	3.4 kb	*Streptococcus thermophiles*	NNAGAAW	[19]
SaCas9	3156 bp	*Staphylococcus aureus*	3’ NNGRRT or NNGRR(N)	[19]
SpCas9 D1135E variant	4104 bp	*Streptococcus pyogenes*	3’ NGG	[19]
SpCas9 VRER variant	4104 bp	*Streptococcus pyogenes*	3’ NGCG	[19]
SpCas9 EQR variant	1716 bp	*Streptococcus pyogenes*	3’ NGAG	[19]
SpCas9 VQR variant	4104 bp	*Streptococcus pyogenes*	3’ NGAN or NGNG	[19]
SpCas9 nickases	4.1 kb	*Streptococcus pyogenes*	Enhanced specificity,3’ NGG	[10]
Dimeric FokI-dCas9 fusions				[21]
NmCas9	3243bp	*Neisseria meningitidis*	NNNNGMTT	[15]
FnCas12a	3.9 kb	*Francisella novicida*	TTN, CTA	[22]
AsCas12a	3921bp	*Acidaminococcus sp.*	5’ TTTV	[23]
AsCas12aRR variant	3918 bp	*Acidaminococcus sp*	*5’ TYCV*	[23]
AsCas12a RVR variant	3918 bp	*Acidaminococcus sp*	5’ TATV	[23,24]
LbCas12a	3684 bp	*Lachnospiraceae bacterium*	5’ TTTV	[23]
LbCas12a RR variant	3681 bp	*Lachnospiraceae bacterium*	5’ TYCV	[23,24]
AacCas12b	3386 bp	*Alicyclobacillus acidoterrestris*	5’ NTTV, VTTTV	[25]
AaCas12b	3486 bp	*Alicyclobacillus acidiphilus*	5’ NTTV, VTTTV	[25]
BthCas12b	3423 bp	*Bacillus thermoamylovorans*	5’ NTTV	[25]
BhCas12b	3423 bp	*Bacillus hisashii*	5’ NTTV	[25]
Cas12e (or DpbCasX)	2958bp	*Deltaproteobacteria*	5’TTCN	[3]
Cas12j (also known as Casɸ)	2142bp	Phage	5’TTA	[4]

**Table 2:** List of different CRISPR/Cas9 vectors for genome engineering in plants.

CRISPR/Cas Vectors	Size (Kb)	Bacterial Selection	Replication Origin Agro, *E. coli*	Plant Selection Gene	Remark	References
**Knock out Vectors**						
pRGEB31	15.0	Kan	pVS1, ColE1	Hyg	Cas9 regulated under CaMV35; gRNA under OsU6	[32]
pHSN401	12.7	Kan	pSA, pUC	Hyg	Cas9 regulated under CaMV35; gRNA under AtU6	[37]
pRGEB32	15.8	Kan	pVS1, ColE1	Hyg	Cas9 regulated with maize Ubi promoter	[36]
pBSN401	12.5	Kan	pSA, pUC	Bar	Cas9 regulated under CaMV35; gRNA under AtU6	[37]
pKSE401	12.5	Kan	pSA, pUC	Kan	Cas9 regulated under CaMV35; gRNA under AtU6	[37]
pHSE401	16.6	Kan	pVS1, ColE1	Hyg	Cas9 regulated under CaMV35; gRNA under AtU6	[37]
pFGC-pcoCas9	13.3	Kan	pVS1, ColE1	Bas	Plant codon optimised Cas9 under a hybrid 35SPPDK promoter	[46]
Cas9 MDC32	14.1	Kan	pVS1, RepA, sta1	Hyg	Soybean codon optimized Cas9 regulated by 35S promoter	[25]
Cas9 MDC123	13.6	Kan	pVS1, RepA, sta1	Bas	Soybean codon optimized Cas9	[25]
G10 Cas9 MDC123	13.2	Kan	pVS1, RepA, sta1	Bas	Soybean codon optimized Cas9 regulated by G10 promoter	[25]
pDIRECT_22C	16.0	Kan	pVS1, ColE1	Kan	Csy4-P2A fused with Arabidopsis codon optimised Cas9	[38]
pGEL031	15.7	Kan	pVS1, ColE1		Cas9 regulated with maize Ubi promoter	[64]
pGEL029	15.6	Kan	pVS1, ColE1		Cas9 regulated with maize Ubi; gRNA without promoter	[64]
pG3H-U6EC1	13.6	Kan	ColE1	Hyg	pGreen3 derived vector, required pSoup vector for *Agrobacterium*	[65]
pAGM51547		Kan	pVS1, ColE1	Bar	Cas9 with multiple introns; incresed editng efficiency	[66]
pAGM55273	17.0	Kan	pVS1, ColE1	Kan	Cas9 with multiple introns; incresed editng efficiency	[66]
**Knock-in Vectors**						
pTC217	17.9	Kan	pVS1, RepA	Kan	Bean Yellow Dwarf Virus replicon based; donor teplated is Pnos:NptII-35S:ANT1 flanked with 5’ and 3’ homology arm	[67]
pDe-Cas9-Hpt-GT-DFR#3-DFR#4-DFRtemp	Greater than 16	Chlor and Spec	pVS1, sta1	Hyg	Donor DNA left and right homologous arms, each corresponding to the 400 bp and 392 bp sequences flanking both sides of the 1013 bp DFR deletion	[68]
TmicT2donorRepUbi10	NA	Kan	NA	Kan	contains the CRISPR/Cas9, Rep protein on the T-DNA outside the geminiviral replicon and the donor repair template is within the replicon.	[69]
TmicT1donorRepUbi10	NA	Kan	NA	Kan	Same as above	[69]
pRGEB-VirD2-Cas9	17.2	Kan	pVS1, ColE1	Hyg	Chimeric Cas9-VirD under ubiquitin promoter; VirD2 relaxase facilitates homology-directed repair	[70]
pTC217 and pTC223	17.9	Kan	pVS1, ColE1	Kan	gRNA targeting ANT1 locus, donor DNA having 5’ homology arm-Pnos:NptII-35S:ANT13’ homology arm, bean yellow dwarf virus replicon	[67]
**CRISPRi Vectors**						
pYPQ153, pYPQ series	7.7	Spec	ColE1	Absent	Plant codon-optimized dCas9 fused with mutated SRDX (X3) repressor	[71]
pHSN6I01	13.0	Kan	pSA, ColE1	Hyg	Maize codon optimized dCas9-KRAB (Krüppel-associated box)	[37]
pdCas9 (GB1079)	7.1	Amp	f1 ori, ColE1	Absent	Cas9 coding region with mutated (D10A, H840A) and inactivated catalytic domains (human codon optimised)	[72]
pDIRECT_21B	15.2	Kan	pVS1, ColE1	2x35S:hpt II	35S:AtCas9_dead + AtU6:gRNA	[38]
pDIRECT_21D	16.2	Kan	pVS1, ColE1		35S:Csy4-P2A-AtCas9_dead + CmYLCV:gRNAs with Csy4 spacers	[38]
pDIRECT_23D	15.7	Kan	pVS1, ColE1	2x35S:bar	35S:Csy4-P2A-AtCas9_dead + CmYLCV:gRNAs with Csy4 spacers	[38]
**CRISPRa Vectors**						
pEGB 35s:dCas:EDLL:tNos (GB1190)	12.1	Kan	pVS1, sta1	Absent	Human codon optimizedinactivated Cas9 fused to the EDLL transcriptional activator	[72]
pYPQ152	7.7	Spec	Absent, ColE1	Absent	Plant codon-optimized dCas9 fused with VP64 activator	[71]
pHSN6A01	13.0	Kan	pSA, ColE1	Hyg	Maize codon-optimized dCas9 fused with VP64 (4× minimal VP16 activation domain)	[37]
pBUN6A11	13.8	Kan	pSA, ColE1	Bar	Maize codon optimized dCas9 fused with VP64 (4× minimal VP16 activation domain)	[37]
**Vectors for Visualization**						
pK7WGF2::hCas9	14.4	Spec	pVS1, ColE1	Kan	human codon usage Cas9 nuclease with an N-terminal GFP tag	[26]
pHAGE-TO-dCas9-3XGFP	12.9	Amp	ColE1	Absent	SpdCas9 fused with 3XsfGFP	[73]
pHAGE-EFS-dCas9-GFP	11.0	Amp	ColE1	Absent	SpdCas9 fused with 3XsfGFP	[73]
NmdCas9-3xGFP		Amp	ColE1	Absent	Chimeric NmdCas9 fused with GFP	[74]
SpdCas9-3xCherry	12.9	Amp	ColE1	Absent	Chimeric SpdCas9 fused with 3xCherry P	[74]
p221z-CAS9p-TagRFP-t35s	7.2	Ble	ColE1	Absent	Chimeric Cas9 fused withTagRFP	[75]
**Nickase Vectors**						
pHSN501	12.7	Kan and Spec	pSA, ColE1	Hyg	CaMV regulated zCas9D10A, gRNA under AtU6-26 promoter	[37]
pBUN501	13.4	Kan and Spec	pSA, ColE1	Bar	Ubi promoter regulated zCas9D10A, gRNA under AtU6-26 promoter	[37]
**Transient Expression Vectors**						
pYPQ166-iSpyMac	7.7	Spec	Absent, ColE1	Absent	Cas9 compatible for NAAR PAM	[76]
pYPQ239-RVR	7.1	Spec		Absent	Rice codon optimized *Francisella tularensis* Cas12a (Cpf1)	[77]
pRGE32	10.0	Amp	pVS1, ColE1	Absent	gRNA under rice snoRNA U3 promoter; Cas9 under with rice ubiquitin promoter	[36]
pRGE31	9.1	Amp	pVS1, ColE1	Absent	gRNA under rice snoRNA U3 promoter	[32]
HBT-pcoCas9 and p35SPPDK-pcoCas9	7.9	Amp	Absent, ColE1	Absent	Hybrid constitutive promoter 35SPPDK regulates Cas9	[46]
**Cloning/Assembly/Entry Vectors**						
p221z-CAS9p-t35s	6.5	Ble	Absent, ColE1	Absent	Based on pDONR-221z	[75]
pYPQ131-STU-Fn	3.1	Tet	Absent, ColE1	Absent	For crRNA cloning flanked by HH and HDV ribozymes; compatible with FnCas12a	[78]
pFH16	6.1	Spec	ColE1	Absent	Rice codon optimized FnCas12a	[79]
pYPQ166-SpRY	7.7	Spec	Absent, ColE1	Absent	Gateway compatible zSpRYCas9 entry clone; PAM less Cas9	[80]
pYPQ239-RR	7.1	Spec	Absent, ColE1	Absent	Entry for FnCas12a	[77]
pMOD_B2101	3.5	Amp	Absent, ColE1		SapI ccdb cassette for cloning multiple gRNA protospacers with Csy4 spacers	[38]
pMOD_B2301	3.6	Amp	Absent, ColE1		SapI ccdb cassette for cloning multiple gRNA protospacers with tRNA spacers	[38]
pMOD_B2403	3.5	Amp	Absent, ColE1		SapI ccdb cassette for cloning multiple gRNA protospacers with ribozyme spacers	[38]
pYPQ230 (LbCpf1)	7.3	Spec	Absent, ColE1/pBR322/pUC	Absent		[81]
pBlu/gRNA	3.5	Amp		Absent	Direct cloning of target oligo duplex	[25]
pYPQ131-138	3.8	Tet	Absent, ColE1	Absent	Golden Gate entry vector; one to eighth gRNA under AtU6 promoter	[71]
pICSL01009	2.3	Spec	Absent, ColE1	Absent		[26]
pYPQ141-148	3.6	Spec	Absent, ColE1	Absent	Golden Gate entry vector; one to eighth gRNA under different U6 promoter	[71]
pYPQ141-ZmUbi-RZ-Lb	4.9	Spec	Absent, ColE1	Absent	Lb Cpf1 gRNA cloning site for ribozyme cleavage	[81]
pCBC-DT1T2	3.5	Chlor	Absent, ColE1	Absent	Cloning of two target under AtU6 promoter	[37]
pEF005-sgRNA-shuffle-in	2.9	Amp	Absent, ColE1	Absent	Provide and shuffle a cassette of AtU6:sgRNA-transRNA into SM-destination vectors (pRW006 and pRW004) with golden gate cloning strategy.	[82]
**Viral Replicon Vectors**						
pTRANS_101	5.8	Spec	Absent, ColE1	Absent	Non-T-DNA None BeYDV	[38]
pTRANS_102	6.2	Spec	Absent, ColE1	Absent	Non-T-DNA None ToLCV	[38]
pTRANS_211	12.4	Spec	pVS1, ColE1	Hyg	Non-T-DNA None WDV	[38]

Kan—Kanamycin; Chlor—Chloramphenicol; Spect—Spectinomycin; PPT—phosphinothricin; Tet—Tetracycline; Ble—Bleocin(Zeocin); NA—not available.

## Data Availability

Not applicable.
